# Camel grazing reshapes root and rhizome economic strategies and trade-offs: synergistic roles of soil water, salinity, and nitrogen availability

**DOI:** 10.3389/fpls.2026.1876859

**Published:** 2026-07-09

**Authors:** Jing Zhang, Kai Yan, Wei Zheng, Yingzhi Gao, Meng Cui, Jia Shaer, Yang Li, Xiang Li, Yerlan Akebieke, Ayiding Hezhati, Lianghong Wu

**Affiliations:** 1 College of Grassland Science, Xinjiang Agricultural University, Urumqi, China; 2 Grassland General Station of Xinjiang Uygur Autonomous Region, Urumqi, China; 3 Xinjiang Key Laboratory of Grassland Resources and Ecology, Urumqi, China; 4 College of Grassland Science, Inner Mongolia Agricultural University, Hohhot, China; 5 Xinjiang Uygur Autonomous Region Grassland Biological Disaster Prevention and Control Center (Regional Wildlife Protection and Monitoring Center), Urumqi, China

**Keywords:** camel grazing, dominant species, functional traits, resource-acquisition strategies, soil heterogeneity

## Abstract

**Introduction:**

Camel grazing drives grassland degradation in arid regions. However, how does it affect the synergistic changes in soil water, salinity, and nitrogen availability? Moreover, how does it reshape the trade-offs between root and rhizome economic strategies? Quantitative studies addressing these questions remain scarce.

**Methods:**

This study investigated saline meadow ecosystems under camel grazing in arid regions, comparing three grazing treatments: no grazing (NG), controlled grazing (CG), and free grazing (FG). Nine soil physicochemical properties and nine root and rhizome functional traits of dominant plants were assessed across three soil layers (0-25, 25-50, and 50–100 cm). The findings provide critical insights to support sustainable camel grazing management in arid salt-affected meadows.

**Results and discussion:**

Camel grazing depletes nitrogen, accumulates salt, and redistributes water in the 0–25 cm soil layer. Consequently, the root functional trait syndrome shifts from acquisitive to conservative along PC1, whereas the rhizome functional trait syndrome shifts from reproductive to storage along PC2. Notably, nonlinear thresholds trigger these shifts, with threshold values of available nitrogen (AN) <42 mg·kg^-^¹, electrical conductivity (EC) >3144 µS·cm^-^¹, and soil water content (SWC) <10%. Grazing influences root and rhizome economic strategies via one direct and three indirect pathways (nitrogen depletion, salt accumulation, water redistribution). Grazing intensity, soil water, and nitrogen jointly filter the root acquisition-conservation axis, whereas salt and nitrogen jointly filter the rhizome reproduction-storage axis. These findings reveal a water-salt-nitrogen tripartite axis governing the differentiation of root and rhizome economic strategies and provide a scientific basis for threshold-based precision camel grazing management.

## 1 Introduction

The grazing activities of large herbivores have profound effects on global grassland ecosystems. This impact is especially pronounced in arid grasslands, where habitats are fragile. Key pathways include vegetation removal, trampling, and dung and urine deposition (Zhurlov, 2023; Kakar, 2025). Previous studies have largely focused on cattle, sheep, and wild herbivores (Trepel et al., 2024). Their impacts on plant functional traits, spatial heterogeneity, and ecological processes are well documented (Díaz et al., 2007; Guo et al., 2025). Camel grazing is common in arid and hyper-arid regions. However, its specific effects have received insufficient attention (Askar et al., 2024; Yan et al., 2024). Camels are widely distributed in arid regions across the globe, including North Africa, the Middle East, Central Asia, and Australia (Faye, 2015). They provide important ecosystem services such as transportation, milk and meat production, and desert ecosystem maintenance (Kakar, 2025). Compared with cattle and sheep, camels have distinct ecological niche differences. They can utilize coarse fibrous plants that other livestock avoid (Iqbal and Khan, 2001; Mohammed et al., 2020), and their unique physiological adaptations allow them to survive in extreme arid conditions. Camels differ from other ruminants in foraging height, selective feeding behavior, broad, padded hoof structure, and the dispersed deposition of salt-rich feces and urine (Mohammed et al., 2020). These characteristics likely create unique spatial patterns of water, salt, and nitrogen availability, exerting distinct influences on root and rhizome response strategies. Therefore, dedicated research is urgently required into how camel grazing reshapes plant belowground strategies through soil heterogeneity.

Plant functional traits link environmental heterogeneity to plant performance (Cornelissen et al., 2003). Subsurface traits are critical for soil resource acquisition and storage (He et al., 2020). Root economy theory proposes a balance between acquisitive and conservative strategies (Díaz et al., 2016). However, belowground trait variation is multidimensional; plant rhizome traits are generally closely associated with clonal reproduction and nutrient storage (Duchoslavová and Jansa, 2018). In arid regions, water and salinity stress, in combination with nutrient scarcity, limit plant growth and community assembly, rendering the rhizome reproduction-storage axis as important as the classic root acquisition-conservation axis (Thakur and Münzbergová, 2022). Existing evidence is derived primarily from sheep and cattle grazed semiarid grasslands. Whether this two-dimensional strategy applies to camel-grazed arid grasslands remains unclear and requires verification.

Crucially, the impact of grazing on soil properties varies systematically with soil depth (Wu et al., 2022). The surface layer is directly exposed to feces, urine, and trampling, whereas the deeper layers are indirectly affected by water infiltration, root channels, and salt leaching (Reay et al., 2023; Kao et al., 2024). Furthermore, in arid ecosystems, vertical gradients in water and salinity are steep, and root systems generally exhibit depth-specific resource-acquisition strategies (Xu, 1998; Wang et al., 2021). Therefore, distinguishing between soil heterogeneity at multiple depths and the corresponding responses in root traits is crucial for elucidating the mechanisms underlying the relationship between camel grazing, soil patch formation, and shifts in underground strategies (Guo et al., 2025; He et al., 2025). However, systematic research is lacking on the effects of camel grazing intensity on the depth dependence of soil water, salinity, and nitrogen, and how this heterogeneity drives underground functional trait differentiation and resource strategy adjustment.

This study investigates how camel grazing intensities (no grazing (NG), controlled grazing (CG), and free grazing (FG) affect root and rhizome functional traits of dominant plants in a saline meadow in arid Xinjiang, China. By integrating soil properties (soil water content (SWC), electrical conductivity (EC), available nitrogen (AN)) and root and rhizome functional traits across three soil depths (0-25cm, 25-50cm, 50–100 cm), focus is placed on elucidating three key scientific questions: (1) What is the change of the principal dimensions of root and rhizome economic strategies under different grazing intensities? What is the contribution of key driving factors? (2) Do these economic strategies show nonlinear threshold responses to SWC, EC, and AN gradients? What are the key ecological processes that the thresholds represent? (3) Are the two principal axes of root and rhizome functional trait-based economic strategies driven by specific soil factors? What is the relative contribution of each factor? The findings will support understanding of grazing-induced belowground processes and sustainable camel grazing management.

## 2 Materials and methods

### 2.1 Study area

The study area is located in Xiasangong Village, Yuanhu Town, Hutubi County, in the southern Junggar Basin, Xinjiang, China (N 44°21′25.41″, E 86°56′42.95″, elevation 368 m). It consists of a typical lowland salt-affected meadow dominated by *Phragmites australis* (Cav.) Trin., serves as a transitional ecosystem between the oasis agricultural zone and the desert. The study area has a temperate continental arid climate. From 2020 to 2024, the average annual precipitation was 154.24 mm, with an average annual temperature of 8.94 °C, an average annual minimum temperature of −27.5 °C, and an average annual maximum temperature of 39.66 °C (**Supplementary Table 1**). The soil is classified as meadow saline soil. Since 1983, when collective pastures were contracted to individual households, the area has been utilized for mixed grazing of sheep, horses, and camels. Since 2011, driven by industrial restructuring, it has been utilized exclusively for camel grazing.

### 2.2 Experiment design

To ensure analytical independence and statistical power, an appropriate study area of 5 hectares was established for each treatment based on camel foraging rates, grassland carrying capacities, and the spatial requirements of camel grazing behavior. The total experimental site covered 15 hectares and was divided via fencing into three treatments: NG, with a 10−year grazing exclusion; CG, with two adult camels and one juvenile camel, 0.5 camels/ha; and FG, with ten camels, 2 camels/ha. Both grazing treatments were applied for 180 days per year, from May to October. Five subplots (0.30 ha each, 15 m × 20 m) were established per treatment. In the CG and FG treatments, subplots were arranged along the grazing pressure gradient from the pens and watering points, at distances of 0, 100, 200, 300, and 400 m (following the “near-to-far, high-to-low” principle). A three−year survey was conducted, during which 75 plots were investigated per treatment, for a total of 225 examined plots. It should be noted that each treatment had only one paddock, and the five sampling points represented spatial replicates along the grazing intensity gradient rather than treatment replicates. Therefore, the subplots should be considered as subsamples nested within sampling locations and paddocks, rather than independent treatment replicates. Soil sampling was conducted using a random sampling method. For rhizome sample selection, only fine roots with a diameter range of 0–2 mm were collected.

### 2.3 Soil sampling and property measurement

Soil samples were collected in mid-July between 2022 and 2024. Because 90% of herbaceous roots occur within 0–100 cm, soil sampling depths were set at 0–25 cm, 25–50 cm, and 50–100 cm (Wang, 2021). Using the excavation method, a total of five 1 m³ soil profiles were randomly dug per plot group. For each depth, 100 cm³ ring cutters collected soil for moisture content and bulk density (BD) (five replicates per group). Then, “mid-layer” soil from the same depth across the five profiles was mixed, sieved (2 mm) to remove debris and gravel, and ~500 g per layer was retained in breathable cloth bags for nutrient analysis. Annually, 75 profiles were surveyed, leading to 225 profiles being excavated over three years, yielding 675 soil samples, of which 270 were sent to the laboratory after mixing and screening. Soil physicochemical properties were determined following the methods described by Bao (2000). Soil organic carbon (SOC) was measured by potassium dichromate oxidation; SWC by oven drying; BD by the ring knife method; EC was measured using a 1:5 soil-to-water extraction ratio at 25 °C; pH by potentiometry; AN by alkaline diffusion; available phosphorus by Olsen method; available potassium by ammonium acetate extraction-flame photometry; and total nitrogen (TN) by Kjeldahl digestion.

### 2.4 Root and rhizome functional trait measurements

*P. australis* is a typical clonal plant. Its rhizome is a core component of nutrient storage and clonal reproduction, as well as a key functional trait in response to soil heterogeneity and grazing disturbance (Xu et al., 2012).

*P. australis* was selected as the dominant species within each plot, and live fine roots of this species were present at every sampling point (Cornelissen et al., 2003). A soil profile of 1 m × 1 m × 1 m was excavated. Within the 1 m³ soil profile described in Section 2.3, the soil was divided into three layers: 0–25 cm, 25–50 cm, and 50–100 cm (Wang, 2021). All belowground organs of *P. australis* were collected separately by soil layer.

Samples were placed in self-sealing bags and stored in a cooler containing ice. Root samples were packed in nylon mesh bags and weighed fresh. Subsequently, they were fully laid out on a whiteboard without overlapping. The board was marked with a scale and sample numbers. Measurements of belowground functional traits followed the international standardized measurement manual (Cornelissen et al., 2003). Dense root samples were arranged on separate trays. A camera (Canon, Tokyo, Japan) was used to acquire clear root images with scale markers. Image analysis was performed using the LA-S root area analyzer system (Wanshen Testing Technology Company Limited, China). Morphological traits were obtained, including root length (RL, cm), rhizome length (RZL, cm), root surface area (RSA, cm²), rhizome surface area (RZSA, cm²), root volume (RV, cm³), rhizome volume (RZV, cm³), and rhizome diameter (RZD, cm). These root morphological traits encompass key dimensions of plant resource-acquisition and spatial expansion (Díaz et al., 2016; He et al., 2020).

After field scanning, belowground organs were placed in nylon mesh bags and air-dried in a spacious, well-ventilated indoor environment. Subsequently, rhizome biomass (RZB, g) was measured (de Bello et al., 2021). Specific rhizome length (SRzL, cm·g^-1^) is calculated by the formula: SRzL = Rhizome length/Rhizome dry weight (Thakur and Münzbergová, 2022).

### 2.5 Data processing and statistical analysis

#### 2.5.1 Impact of grazing on soil heterogeneity and the heterogeneity of underground functional traits of dominant species

Linear mixed models with paddock as a random intercept were used for statistical analysis. For each soil physicochemical property and belowground functional trait, and within each soil horizon separately, we fitted a linear mixed model with treatment (NG, CG, FG) as a fixed effect and subplot as a random intercept. Least−squares means (emmeans) were estimated for each treatment, and all pairwise comparisons (NG vs. CG, NG vs. FG, CG vs. FG) were performed using the emmeans package with Tukey adjustment for multiple comparisons. To account for multiple testing across different indicators, the false discovery rate (Benjamini-Hochberg method) was further applied within each soil horizon. Significance levels were denoted as: *P* < 0.05, *P* < 0.01, *P* < 0.001; nonsignificant differences were marked as ns. Grid bar charts were generated using the ggplot2 package.

To quantify the effects of each treatment relative to the control (NG), the log response ratio (ln*RR*) was calculated for each indicator, soil layer, and treatment (CG and FG) (Jiang et al., 2023). For each group, the mean (*X̄*) and standard deviation (S) were calculated. lnRR is defined as ln(*X̄*_t_/*X̄_c_*). The sample variance of ln*RR* is estimated as *S*_t_²/(*n_t_* × *X̄_t_*²) + *S_c_*²/(*n_c_* × *X̄_c_*²). The SE of ln*RR* is the square root of this variance. S_t_ and S_c_ represent the standard deviations of the samples in the treatment group and control group, respectively; *n_t_* and *n_c_* represent the sample sizes of the treatment group and control group, respectively; *X_c_* and *X_t_* represent the means of the treatment group and control group, respectively. The approximate 95% confidence interval is calculated as ln*RR* ± 1.96 × SE. A two-tailed z-test is used to evaluate whether ln*RR* is significantly different from zero (H0: ln*RR* = 0); the p-value is derived from the standard normal distribution. Significance levels are denoted as: *** *P* < 0.001, ** *P* < 0.01, * *P* < 0.05; nonsignificant results are marked as ns. A forest plot visualizing the response ratios was generated using the ggplot2 package.

#### 2.5.2 Relationship between soil heterogeneity and variation in subsurface traits of dominant species

Principal component analysis (PCA) was performed on underground functional traits (RZD, RL, RSA, RV, etc.) of dominant species (Li and Prentice, 2024; Ma et al., 2024). Trait data were centered and standardized. Environmental factors (SWC, EC, and AN) were projected onto the ordination as vectors after correlation with PC1/PC2 scores. Several tests were conducted: (1) PERMANOVA (Bray-Curtis distance) to assess overall trait composition differences among NG, CG, and FG (Rauber et al., 2021); (2) envfit analysis to assess the explanatory power of SWC, EC, and AN on trait ordination (Lins et al., 2021); (3) one−way ANOVA with Tukey’s HSD to compare PC1/PC2 scores among treatments.

To examine nonlinear responses of PC1/PC2 to environmental gradients, generalized additive models (GAMs) and segmented regression were used (Li and Prentice, 2024; Zheng et al., 2026a). GAMs were fitted as *y* ~ s(*x*) with thin−plate regression splines (bs = “tp”) and REML smoothing parameter selection (maximum k = -1). Adjusted *R*² and bias−corrected explanatory power were calculated to evaluate model fit. Thresholds of PC1/PC2 shifts along SWC, EC, and AN gradients were identified based on the GAM and segmented regression outputs.

#### 2.5.3 Structural equation modeling analysis of relationships among grazing intensity, soil heterogeneity, and the belowground traits of dominant species

To investigate the direct and indirect pathways through which camel grazing influences underground functional trait strategies via soil environmental factors, we conducted the following analysis. Grazing intensity was treated as categorical, with no grazing (NG) as the reference, and two dummy variables (CG and FG) were created. All continuous variables were standardized. Path analysis using ordinary least squares (OLS) regression were run using ordinary least squares: SWC, EC, and AN on the two dummies, and PC1 and PC2 on SWC, EC, and AN. Standardized coefficients are reported as path coefficients. Multicollinearity was assessed via variance inflation factors (VIF < 5). Nonparametric bootstrapping (1,000 resamples) provided robust standard errors, 95% percentile confidence intervals, and *P*-values (normal approximation, *α* = 0.05). Model explanatory power was evaluated with R² for each endogenous variable.

## 3 Results

### 3.1 Changes in soil physicochemical properties under different grazing intensities

**Figure 1:**
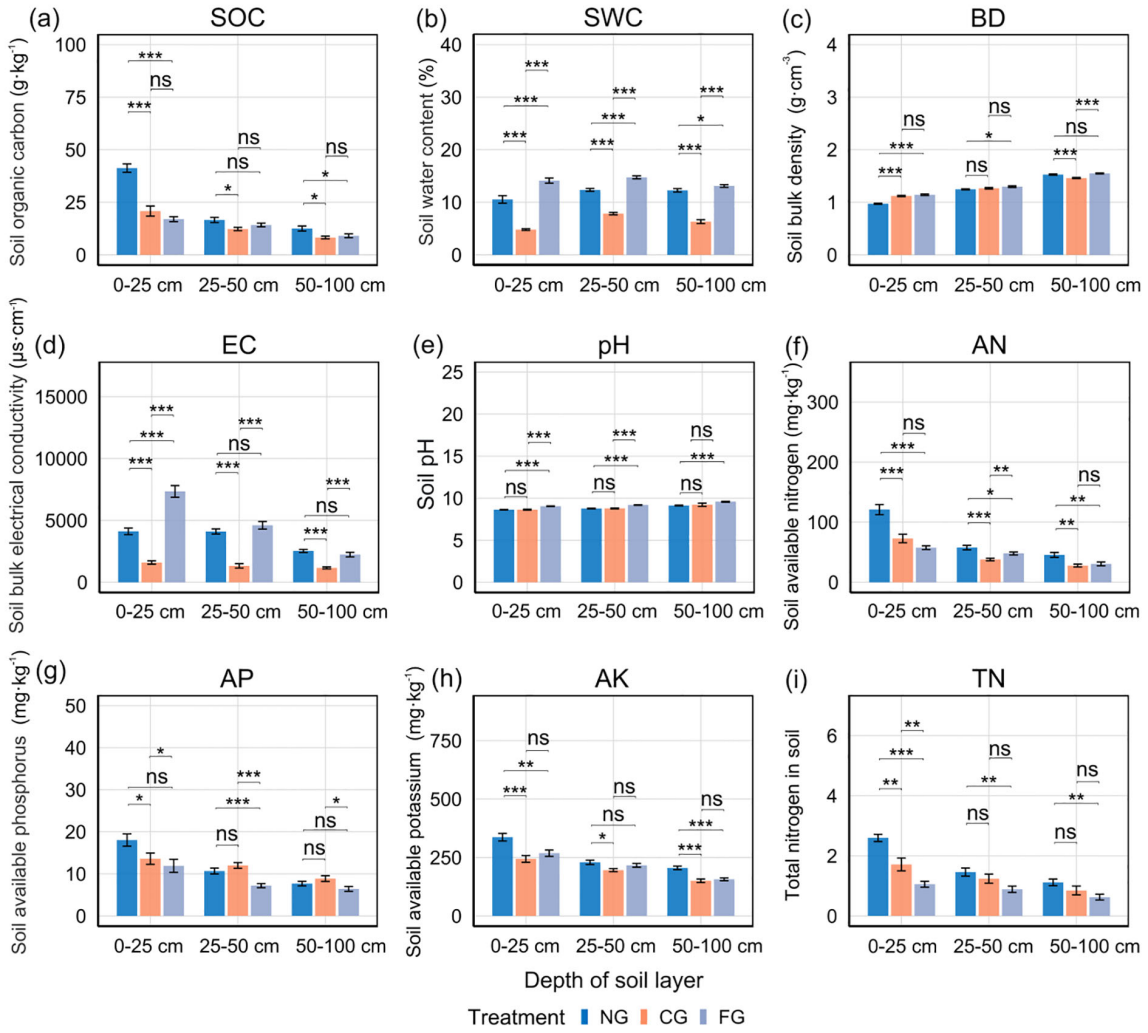
Impact of camel grazing intensity on the physicochemical properties of soil layers. **(A)** Soil organic carbon, **(B)** soil water content, **(C)** bulk density, **(D)** electrical conductivity, **(E)** pH, **(F)** available nitrogen, **(G)** available phosphorus, **(H)** available potassium, **(I)** total nitrogen. In different treatments, NG represents the no-grazing control group, CG represents the controlled-grazing group, and FG represents the free-grazing group. * *P* < 0.05, ** *P* < 0.01, *** *P* < 0.001; ns = significant difference.

Grazing intensity marked altered SOC as well as moisture, salinity, alkalinity, and nutrient content, with variations observed across soil depth (**Figure 1**). Among these, SWC, EC, and AN exhibited more pronounced differences vertically within the soil profile and horizontally across treatment levels. SWC was highest in the FG treatment, lowest in the CG treatment, and intermediate in the NG treatment across all soil layers, with differences among treatments (*P* < 0.05; **Figure 1B**). Soil: Exhibited a pattern of salt accumulation in the surface layer (**Figure 1D**). In the 0–25 cm soil layer, soil EC in the FG treatment was higher than in the NG and CG treatments (*P* < 0.001); in the 25–50 cm and 50–100 cm soil layers, there was no difference between the FG and NG treatments, but EC was higher than that of the CG treatment (*P* < 0.001). The response of soil AN to camel grazing exhibited a pattern of “suppression in the surface layer and complexity in the deeper layers” (**Figure 1F**). AN was highest in the NG treatment in the 0–25 cm soil layer, higher than in the CG and FG treatments (*P* < 0.001); however, in the 25–50 cm soil layer, only the NG treatment was higher than the FG treatment (*P* < 0.01), whereas in the 50–100 cm soil layer, there was no difference between the NG and FG treatments, nor the CG and FG treatments.

### 3.2 Changes in underground functional traits of dominant species under various grazing intensities

**Figure 2:**
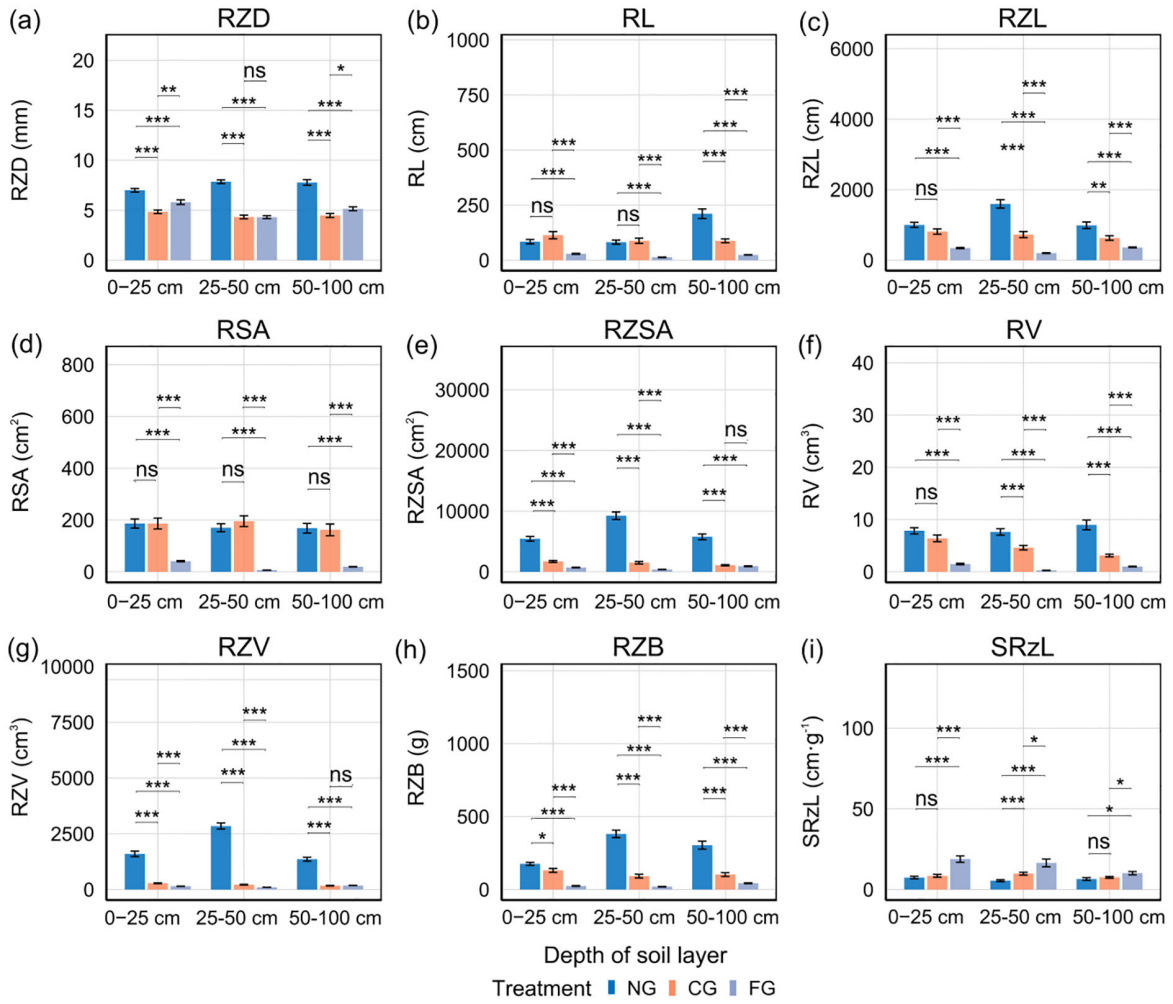
Impact of camel grazing intensity on the spatial heterogeneity of functional traits at various underground depths. **(A)** rhizome diameter, **(B)** root length, **(C)** rhizome length, **(D)** root surface area, **(E)** rhizome surface area, **(F)** root volume, **(G)** rhizome volume , **(H)** rhizome biomass, **(I)** Specific rhizome length. * *P* < 0.05, ** *P* < 0.01, *** *P* < 0.001; ns = significant difference.

Grazing intensity substantially modified the functional traits of roots and rhizomes in dominant species, and these modifications differed across soil depths (**Figure 2**). RZD was highest under NG across all soil layers, higher than under CG and FG, and exhibited a U-shaped pattern (**Figure 2A**). SRzL increased linearly with grazing intensity (*P* < 0.001, **Figure 2I**). RL was highest in the 0–25 cm and 25–50 cm soil layers under NG and CG, higher than under FG; in the 50–100 cm layer, it decreased with increasing grazing intensity (*P* < 0.001, **Figure 2B**). RZL, RSA, RZSA, RV, RZV, and RZB all decreased linearly with increasing grazing intensity, generally following the order NG > CG > FG (**Figures 2C–H**). Specifically, RL and RV in the 0–25 cm layer showed no difference between NG and CG (**Figures 2B, F**), while RSA and RV in the 50–100 cm layer showed no difference between CG and FG (**Figures 2D, F**).

### 3.3 Response effects of soil physicochemical properties and subsurface traits of dominant species to grazing intensity

**Figure 3:**
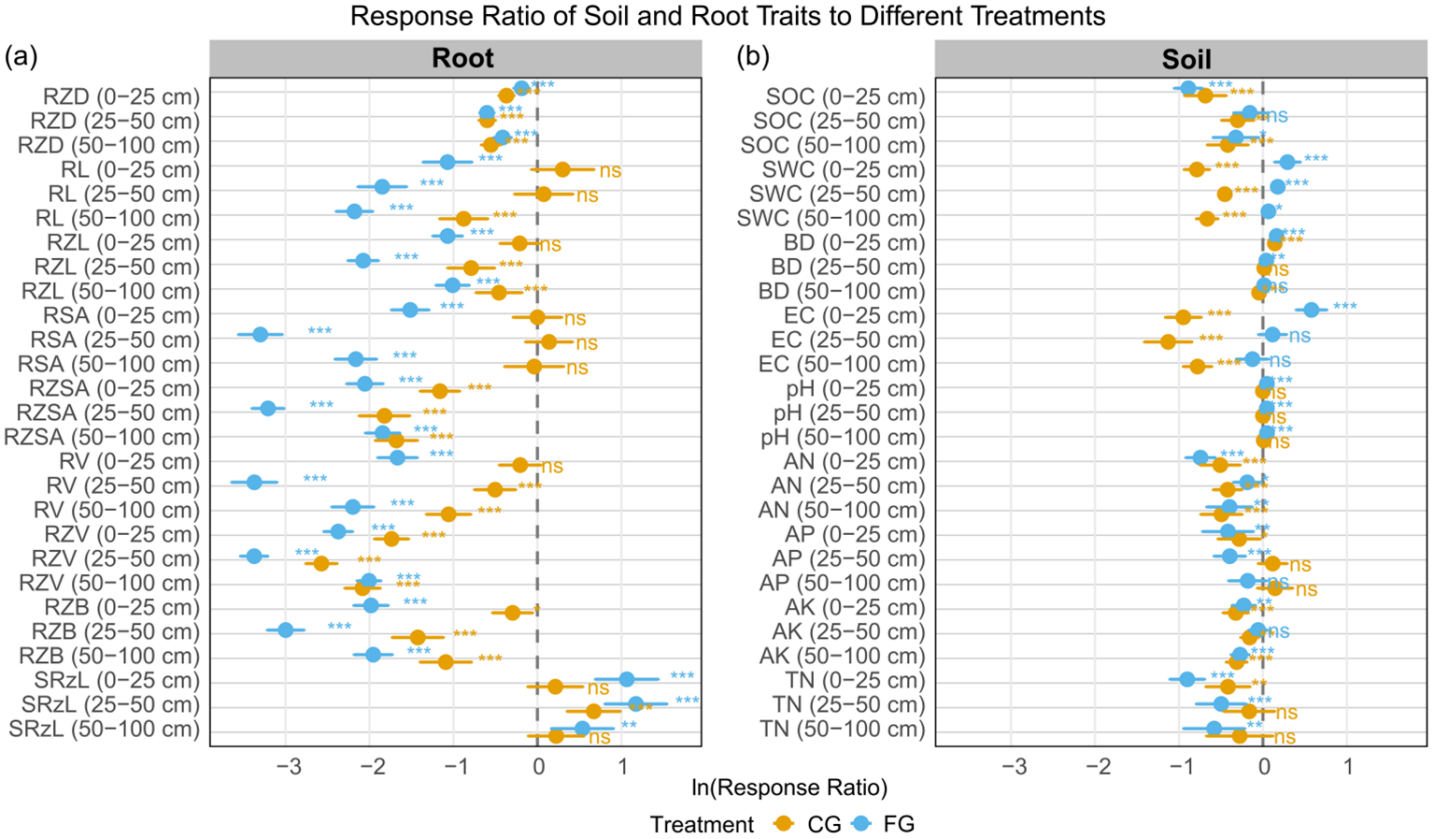
Response effects of soil physicochemical properties and underground functional traits to different grazing intensities. **(A)** Response Ratio of belowground functional traits to grazing, **(B)** Response ratio of soil physicochemical properties to grazing. The dots represent the estimated lnRR values, and the horizontal error lines indicate the 95% confidence intervals. Treatment groups are distinguished by different colors (CG: orange; FG: blue). * *P* < 0.05, ** *P* < 0.01, *** *P* < 0.001; ns = significant difference.

Response ratio analysis indicated a dual asymmetry in the effects of camel grazing. Specifically, the impacts on root and rhizome functional traits were more pronounced among dominant species than among soil physicochemical properties (**Figure 3**). Among these, only SRzL showed a consistent positive response, indicating that camel grazing drives the dominant species toward a conservative, stress-avoidance strategy characterized by low input and high specific surface area (**Figure 3A**). Soil properties exhibited a “sensitive in the surface layer, insensitive in the deep layer” pattern: SOC, AN, and TN decreased linearly with grazing intensity in the surface layer, with a marked interaction between grazing intensity and soil layers for AN. SWC showed a unique bidirectional response, being negative under the CG treatment and positive under the FG treatment. EC decreased substantially in the surface layer under the CG treatment and increased markedly under the FG treatment, confirming that FG induces salt accumulation in the surface layer (**Figure 3B**). Overall, the magnitude of responses in the functional traits of dominant species was generally greater than that of soil physicochemical properties. Notably, CG is not merely an intermediate state; it exerts unique regulatory effects on specific functional dimensions, while the deep soil system exhibits a stronger buffering capacity against camel grazing disturbances.

### 3.4 Strategic shifts in functional traits of superior varieties along gradients of SWC, EC, and AN

**Figure 4:**
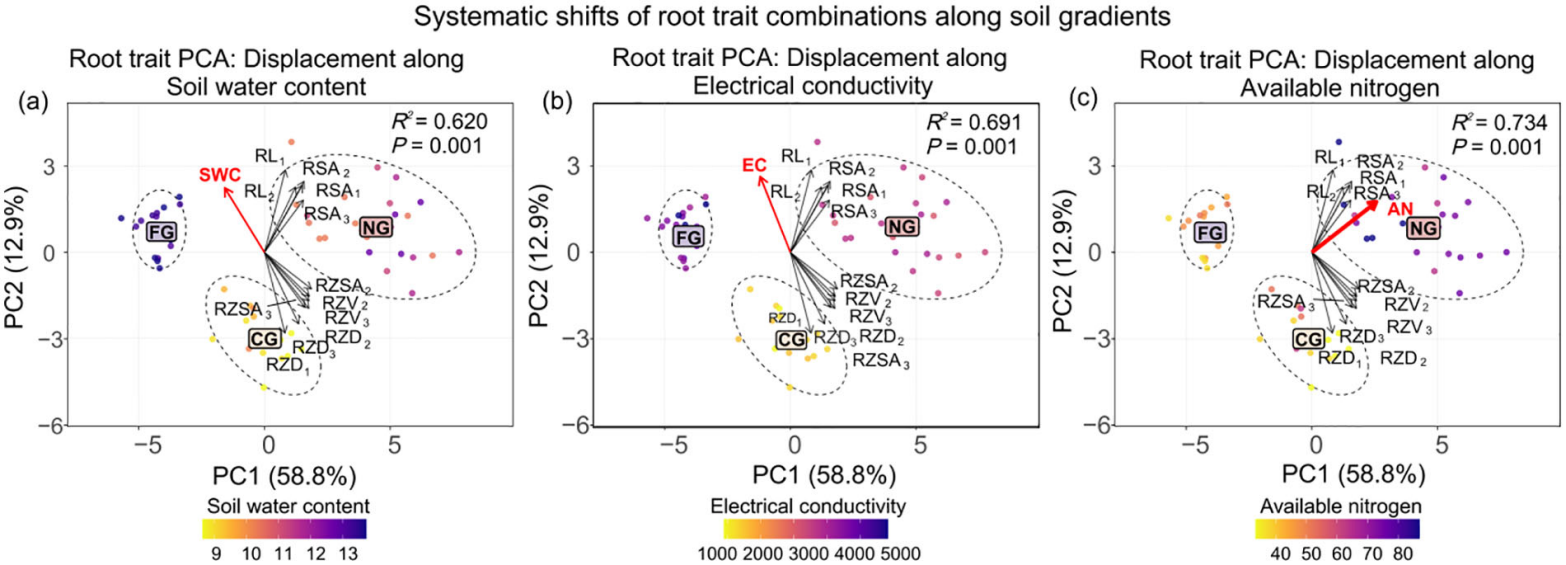
PCA biplots of root traits showing strategy shifts along the SWC, EC, and AN gradients. **(A)** PCA biplots of root traits showing strategy shifts along Soi lwater content, **(B)** PCA biplots of root traits showing strategy shifts along Electrical conductivity, (C) PCA biplots of root traits showing strategy shifts along Available nitrogen. **(A–C)** Samples are colored according to SWC, EC, and AN (low values: yellow-orange, high values: blue-purple); gray arrows indicate the functional trait loadings of dominant species; black arrows represent environmental factor vectors; gray dashed ellipses show the 95% confidence ellipses for each treatment group (NG, No grazing; CG, Controlled grazing; FG, Free grazing).

PERMANOVA based on the Bray-Curtis distance showed that grazing intensity altered the overall composition of root and rhizome functional traits (**Supplementary Table 2**; *R*² = 0.845, *F* = 168.76, *P* = 0.001), indicating distinct trait assemblies among treatments (**Supplementary Table 3**). PCA further illustrated that this shift occurred systematically along soil environmental gradients (**Figure 4**). The first two PCs explained 71.7% of the trait variation. PC1 (58.8%) reflected the grazing intensity gradient (NG → FG). PC2 (12.9%) captured the specific effect of CG. ANOVA confirmed differences for PC1 and PC2 (*P* < 0.001). Tukey’s *post hoc* test showed that along PC1 (root acquisitive-conservative axis), all treatments differed (NG > CG > FG; *P* < 0.001), indicating a progressive shift in traits with increasing grazing intensity. Along PC2 (rhizome reproduction-storage axis), CG scored lower than NG and FG (*P* < 0.001), with no difference between NG and FG (*P*> 0.05), indicating that CG occupies a unique niche rather than an intermediate state.

Envfit analysis (**Supplementary Table 4**, *P* = 0.001) revealed that AN, EC, and SWC were highly associated with trait distribution, with explanatory powers following the order: AN (*R*² = 0.734) > EC (*R*² = 0.691) > SWC (*R*² = 0.620). Along AN (**Figure 4C**), NG sites clustered at the high-AN end, correlating with acquisition-related traits (RL, RSA). Along the EC (**Figure 4B**), FG sites were concentrated at the high-EC end, while CG sites were associated with structural traits (RZD, RZV). Along the SWC (**Figure 4A**), FG sites were distributed in high SWC areas, suggesting that FG enhances water-holding capacity.

### 3.5 Threshold responses of optimal subsoil economic strategies to gradients in soil moisture, EC, and AN

**Figure 5:**
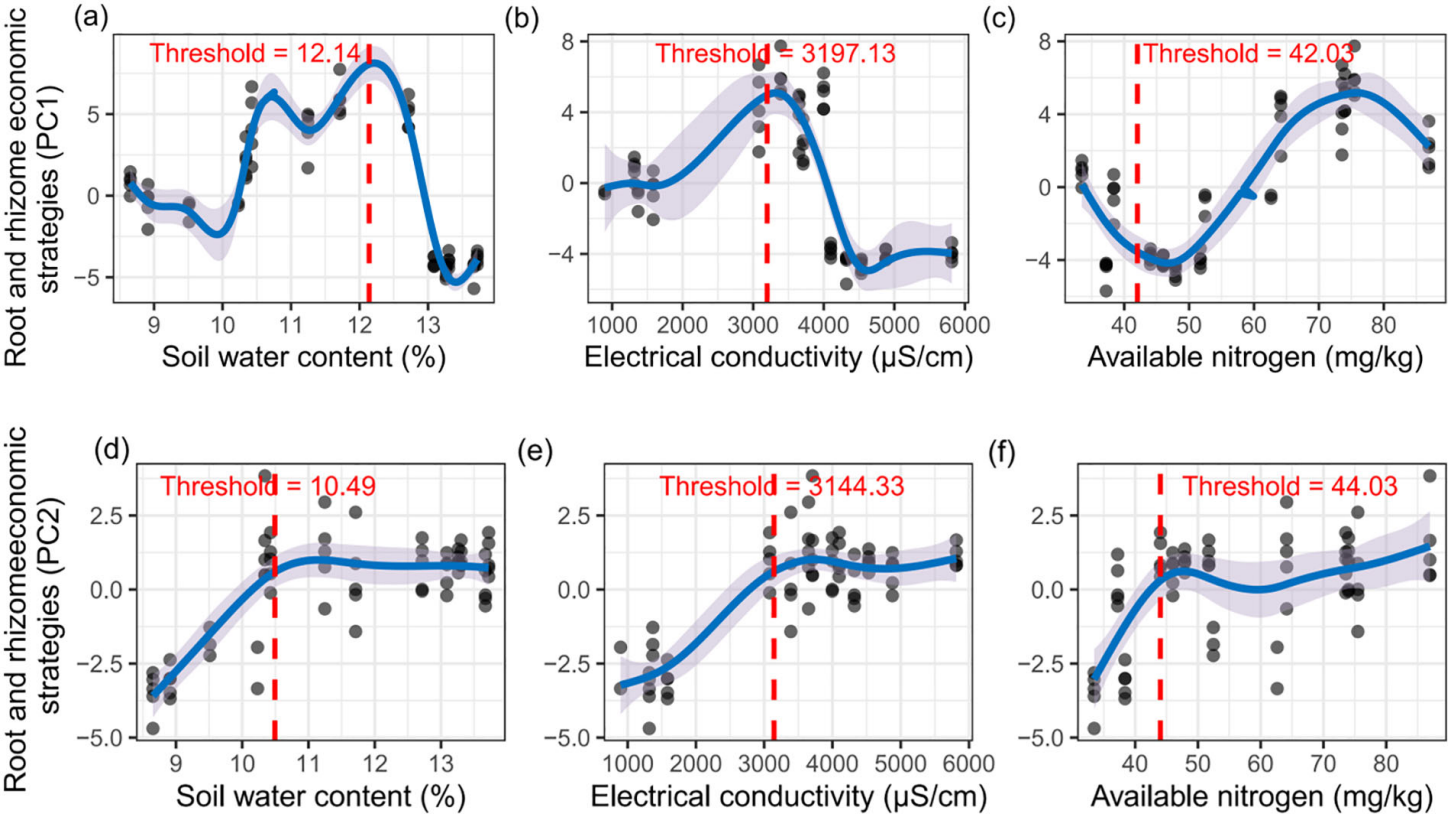
Threshold responses of root economic strategies along the SWC, EC, and AN gradients. **(A)** Response of PCA1 scores of underground functional traits of dominant species to soil moisture gradient, **(B)** Response of PCA1 scores of underground functional traits of dominant species to soil electrical conductivity gradient, **(C)** Response of PCA1 scores of underground functional traits of dominant species to soil available nitrogen gradient, **(D)** Response of PCA2 scores of underground functional traits of dominant species to soil moisture gradient, **(E)** Response of PCA2 scores of underground functional traits of dominant species to soil electrical conductivity gradient, **(F)** Response of PCA2 scores of underground functional traits of dominant species to soil available nitrogen gradient. The blue solid line is the smoothed curve obtained from the GAM fit, with the gray band indicating the 95% confidence interval; the red dashed line marks the marked breakpoints (thresholds) detected by piecewise regression.

Using GAMs and piecewise regression, important ecological thresholds of soil factors driving shifts in belowground economic strategies of dominant species were identified (**Figures 5A, C**). SWC exhibited a critical threshold at 12.14% with a hump-shaped response: below the threshold, root economic strategies tended toward conservatism, while when moderately above this threshold, they shifted rapidly to an acquisitive functional trait syndrome and then reverted when SWC became too high. The EC threshold was 3197 µS·cm^-^¹. Above this, high salinity triggered an abrupt shift from an acquisitive to a conservative functional trait syndrome. The AN threshold was 42.03 mg·kg^-^¹, below it, economic strategies shifted toward a conservative functional trait syndrome, while above it, they shifted toward an acquisitive functional trait syndrome. Akaike information criterion (AIC) values of segmented regression models for all three factors were significantly lower than those of linear models, and likelihood ratio tests were highly significant (**Supplementary Table 5**, *P* < 0.001).

PC2 threshold analysis revealed soil boundaries for specific functional trait syndrome shifts in camel grazing (**Figures 5D–F**). Significant thresholds for SWC, EC, and AN were 10.49%, 3144.33 µS·cm^-^¹, and 44.03 mg·kg^-^¹, respectively, and segmented models had significantly lower AIC values than linear models (**Supplementary Table 4**, *P* < 0.001). Below these thresholds, economic strategies shifted toward the negative (conservative) PC2 direction, whereas above these thresholds, they rapidly shifted toward acquisitive economic strategies and approached saturation. Compared with PC1, the SWC and AN thresholds on PC2 were slightly shifted, indicating independent sensitivity in the grazing-specific dimension. The EC thresholds showed high consistency with those of PC1.

### 3.6 Factors and mechanisms influencing the subsurface tactics of dominant species under camel grazing disturbance

**Figure 6:**
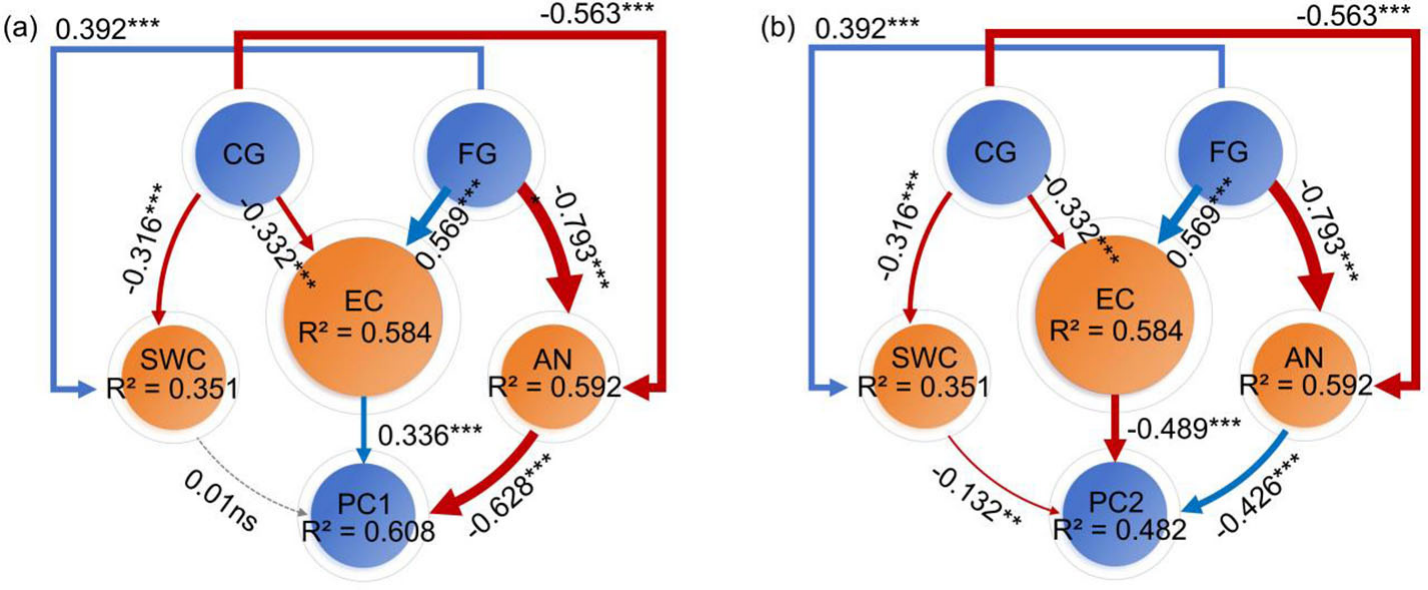
OLS path analysis diagrams. **(A)** The direct and indirect effects of camel grazing intensity on root economic strategies (PC1) via soil variables, **(B)** The direct and indirect effects of camel grazing intensity on root economic strategies (PC2) via soil variables. Grazing intensity was treated as a categorical variable with three levels: NG, CG, and FG. Two dummy variables (CG and FG) were created with NG as the reference. Standardized path coefficients (β) from ordinary least squares regressions are shown next to arrows; solid arrows indicate significant paths (*P* < 0.05, based on bias‑corrected bootstrap 95% confidence intervals, 1,000 resamples), dashed arrows indicate non‑significant paths (ns). Values in parentheses are bootstrap standard errors. The explained variance (R^2^) of each endogenous variable (SWC, EC, AN, PC1, PC2) is given inside the corresponding node. PC1 and PC2, first two principal components of root traits. Significance codes: **P < 0.01, ***P < 0.001; ns, not significant.

OLS path analysis regression revealed that camel grazing indirectly shapes the belowground functional trait strategies of dominant species through SWC, EC, and AN (**Figure 6**). Because grazing intensity was treated as a categorical variable, the effects of moderate (CG) and heavy (FG) grazing were estimated separately. Compared with NG, CG significantly decreased SWC (*β* = –0.32, *P* < 0.001) and EC (*β* = –0.33, *P* < 0.001) and strongly reduced AN (*β* = –0.56, *P* < 0.001). In contrast, FG significantly increased SWC (*β* = 0.39, *P* < 0.001) and EC (*β* = 0.57, *P* < 0.001) but further decreased AN (*β* = –0.79, *P* < 0.001). The soil variables explained 35.1%, 58.4%, and 59.2% of the variance in SWC, EC, and AN, respectively.

In the PC1 submodel (*R*² = 0.608), PC1 was positively driven by EC (*β* = 0.34, *P* < 0.001) and negatively driven by AN (*β* = –0.63, *P* < 0.001), whereas the direct effect of SWC was negligible (*β* = 0.01, *P* = 0.76). In the PC2 submodel (*R*² = 0.399), all three soil variables negatively influenced PC2, with EC (*β* = –0.49, *P* < 0.001) and AN (*β* = –0.43, *P* < 0.001) showing the strongest effects; SWC had a smaller but significant negative effect (*β* = –0.13, *P* = 0.003). No direct paths from grazing to PC1 or PC2 were included in the model; all grazing effects were indirect via the soil variables.

Strong correlations were observed among soil properties: SWC was correlated with EC (*r* = 0.82, *P* < 0.001) and AN (r = 0.72, *P* < 0.001), and EC was correlated with AN (*r* = 0.79, *P* < 0.001) (**Supplementary Table 6**). All variance inflation factors remained below 1.5, indicating no multicollinearity (**Supplementary Table 7**). These results demonstrate that camel grazing alters root economic strategies primarily by modifying soil resources, with moderate and heavy grazing exerting contrasting effects on moisture and salinity but consistent negative impacts on nitrogen availability.

## 4 Discussion

### 4.1 Heterogeneous responses of soil physicochemical properties and deep filtration effects under camel grazing disturbance

Grazing influences soil chemical cycling through mechanisms such as the removal of plant biomass, the input of manure, and changes in the microenvironment (Kao et al., 2024; He et al., 2025). In this study we observed that camel grazing does not exert a uniform degradative effect on soil physicochemical properties; however, it shows marked directional differentiation and depth-dependent effects. SOC and nutrient contents decreased linearly with increasing grazing intensity, and the differences among treatments diminished with increasing soil depth (**Figures 1A, F–I**). This result is consistent with the findings of most previous studies on grazing in arid and semiarid grasslands (Gao et al., 2004). This is closely related to the fact that camels consume and remove large amounts of aboveground biomass, resulting in annual net nutrient losses from the ecosystem exceeding the amount returned to the soil via excreta, thereby creating a long-term net nutrient deficit (Trepel et al., 2024). However, the responses of SWC and EC challenge the conventional wisdom that “grazing homogenization degrades soil quality” (**Figures 1B, D**). CG reduced SWC, whereas FG actually increased SWC, while EC was markedly enriched only in the surface layer under FG. The increase in SWC under FG may result from reduced evapotranspiration resulting from lower vegetation cover, as well as from reduced deep percolation loss of water caused by soil compaction from camel trampling; this compaction, in combination with the special physical structure of the saline-alkali soil profile, leads to higher water-holding capacity. Camels have special physiological characteristics that differ from hoofed livestock. Their broad, padded hoof structure reduces soil penetration resistance (Wilson, 1984; Kakar, 2025), and their unique urination and defecation patterns result in dispersed deposition of salt-rich feces and urine (Faye, 2015; Kao et al., 2024). These characteristics may specifically drive the observed “depth-dependent” effects or salinity accumulation. Compared with cattle grazing in similar arid regions, camels may have a different impact on soil properties. For example, a meta-analysis of cattle grazing in arid regions found that cattle trampling led to more severe soil compaction and reduced water infiltration (Gao et al., 2004; Ming et al., 2024). In contrast, camels’ hoof structure may reduce the degree of soil compaction, allowing for better water retention (Iqbal and Khan, 2001). Depth gradient analysis further indicates that the 0–25 cm surface layer is most sensitive to camel grazing (**Figures 1**, **3**), confirming the depth-filtering effect of the soil system, in which deep soil partially shields grazing disturbance signals through physical buffering and chemical stabilization mechanisms (Ramesh et al., 2024). This pattern of “reshaping of surface-layer heterogeneity and maintenance of deep-layer functions” suggests that grazing management should focus on soil health thresholds in the active rhizosphere layer (Trepel et al., 2024; Li et al., 2025a).

### 4.2 Threshold responses of functional traits across different dimensions in plant-soil feedback

Plant-soil feedback theory posits that plants and soils shape each other bidirectionally (Yacine et al., 2024). This study provides empirical evidence for the “soil selects plant” mechanism by which soil factors actively filter belowground trait strategies via ecological thresholds, yielding a two-dimensional trait space (Lachaise et al., 2022). PC2, a “structure-acquisition” trade-off dimension, responds to soil factors independently of PC1 (the “acquisition-conservation” gradient; **Figure 4**). Using GAM and segmented regression, the thresholds driving strategy shifts in dominant species we quantified as SWC 10.49-12.14%, EC 3144-3197 µS·cm^-^¹, and AN 42.03-44.03 mg·kg^-^¹ (**Figure 5**). These thresholds drive more complex shifts along PC1 than PC2. PC1 captures the main economic spectrum (Gong et al., 2025), involving coordinated adjustments of multiple root traits (Díaz et al., 2016; He et al., 2020). PC2 reflects a trade-off between rhizome structural traits and root acquisition traits. Specific trait dimensions differ in sensitivity to soil factors (Lachaise et al., 2022), such that a smaller threshold shift is required to act on PC2. Notably, the SWC and AN thresholds along PC2 shifted relative to those along PC1 (**Figure 5**), indicating altered plant sensitivity on the grazing-specific dimension. Grazing directly affects root strategies more strongly than soil nutrients (Cai et al., 2024); hence, grazing amplifies plant responses to shifting soil resources.

These findings align with ecosystem state-transition theory (Briske et al., 2005). Previous studies identified grazing thresholds via aboveground cover-erosion relationships (Li et al., 2023). In contrast, in this study, these relationships are extended to belowground functional traits of dominant species (Chillo and Ojeda, 2014). Notably, when the AN content reduces below 42 mg·kg^-^¹, plants abandon acquisition strategies requiring high biomass input, consistent with global nitrogen limitation (Li et al., 2025b; Zheng et al., 2026b). However, unlike traditional saturating nitrogen-response models (Peng et al., 2020), grazing disturbance renders nitrogen effects to exhibit “critical collapse” (Zheng et al., 2026b). Thus, the strategy remains conservative below the threshold and only recovers to an acquisitive state when nitrogen exceeds it (Qiao et al., 2025). The saline-alkali soil profile impedes water-salt movement (Rengasamy, 2010), homogenizing plastic expression in the PC2 space and weakening the driving force of soil factors on this dimension. The selective foraging of camels alters root exudates, modulating deep-root plasticity. Camel urine and feces are deposited in random patches or linear foraging paths (Reay et al., 2023), creating heterogeneous nutrient availability in the 25–50 cm layer and driving PC2-specific responses. Consequently, the non-intermediate state of CG manifests PC2 as an independent dimension. In the 25–50 cm layer, CG maintained root absorbance, partial RL, and RSA at levels similar to or even exceeding those under NG (**Figures 2B, D**). This likely reflects a “moderate disturbance optimization effect” (Kang et al., 2024), extending the moderate disturbance hypothesis from aboveground community structure to belowground functional traits.

### 4.3 Camel grazing reshapes root resource acquisition and rhizome nutrient storage trade-offs via soil factors

This study reveals that salinity and nutrient stress have been underestimated as drivers in grazing ecosystems (Rengasamy, 2010; Salvucci et al., 2023). Traditional research on arid and semi-arid grasslands has generally regarded soil moisture as the primary abiotic limiting factor (Ming et al., 2024). A selection of scholars have noted that “traditional studies have indeed treated soil moisture as the primary abiotic limiting factor, which may have even narrowed the scope of research” (Austin, 2011). The OLS path analysis using ordinary least squares regression shows that grazing indirectly shapes the economic strategies of dominant species’ roots and rhizomes by altering SWC, EC, and AN (Díaz et al., 2007; He et al., 2025). Principal component PC1 is directly suppressed by grazing (Cai et al., 2024) but is indirectly promoted through SWC and AN. EC plays an indirect role by coupling these variables. PC2 is indirectly suppressed by grazing through EC and AN (Trepel et al., 2024; Qiao et al., 2024).

It is important to acknowledge the role of clonal integration in *Phragmites australis*, which may buffer or mask local soil signals. Clonal plants can translocate resources such as water, nutrients, and photosynthates between interconnected ramets through rhizomes (Song et al., 2013), allowing them to maintain physiological function even in heterogeneous environments (Duchoslavová and Jansa, 2018). This means that local root traits may not always directly reflect local soil conditions, as resources can be shared within the clonal network. Indeed, clonal integration can alter root and/or foraging responses of clonal plants when they grow in heterogeneous environments (Roiloa et al., 2010; Thakur and Münzbergová, 2022). In our study, the observed root trait patterns along the grazing gradient may therefore be influenced by both local soil conditions and resource translocation from less stressed ramets. Future studies could use isotopic labeling or split-root experiments to quantify the extent of clonal integration and its effects on root trait expression in grazed ecosystems (Liu et al., 2017).

Strong covariation among soil properties underpins these indirect grazing effects (**Figure 6**). This suggests that grazer-induced edaphic changes do not occur independently of one another. Instead, they propagate through soil networks (He et al., 2025), and ultimately shape belowground economic strategies. The direct negative effects of EC and AN on PC2 align with the stress-gradient hypothesis (Zhang and Tielbörger, 2020), in which increased edaphic stress imposes additional selective filters on stress-tolerant traits (Pavanetto et al., 2025). Elevated salinity and nutrient depletion impose additional filters on stress-tolerant traits. Notably, camel grazing has no direct effect on PC2. This emphasizes that indirect soil pathways can supersede direct herbivore impacts in certain functional dimensions (Pérez-Ramos et al., 2019). Collectively, these findings indicate a need for integrating multi-stressor frameworks, including salinization and N-mining, into predictive models of rangeland functioning under changing land use and climate (Reich, 2014).

### 4.4 Theoretical developments, methodological innovations, and management implications

**Figure 7:**
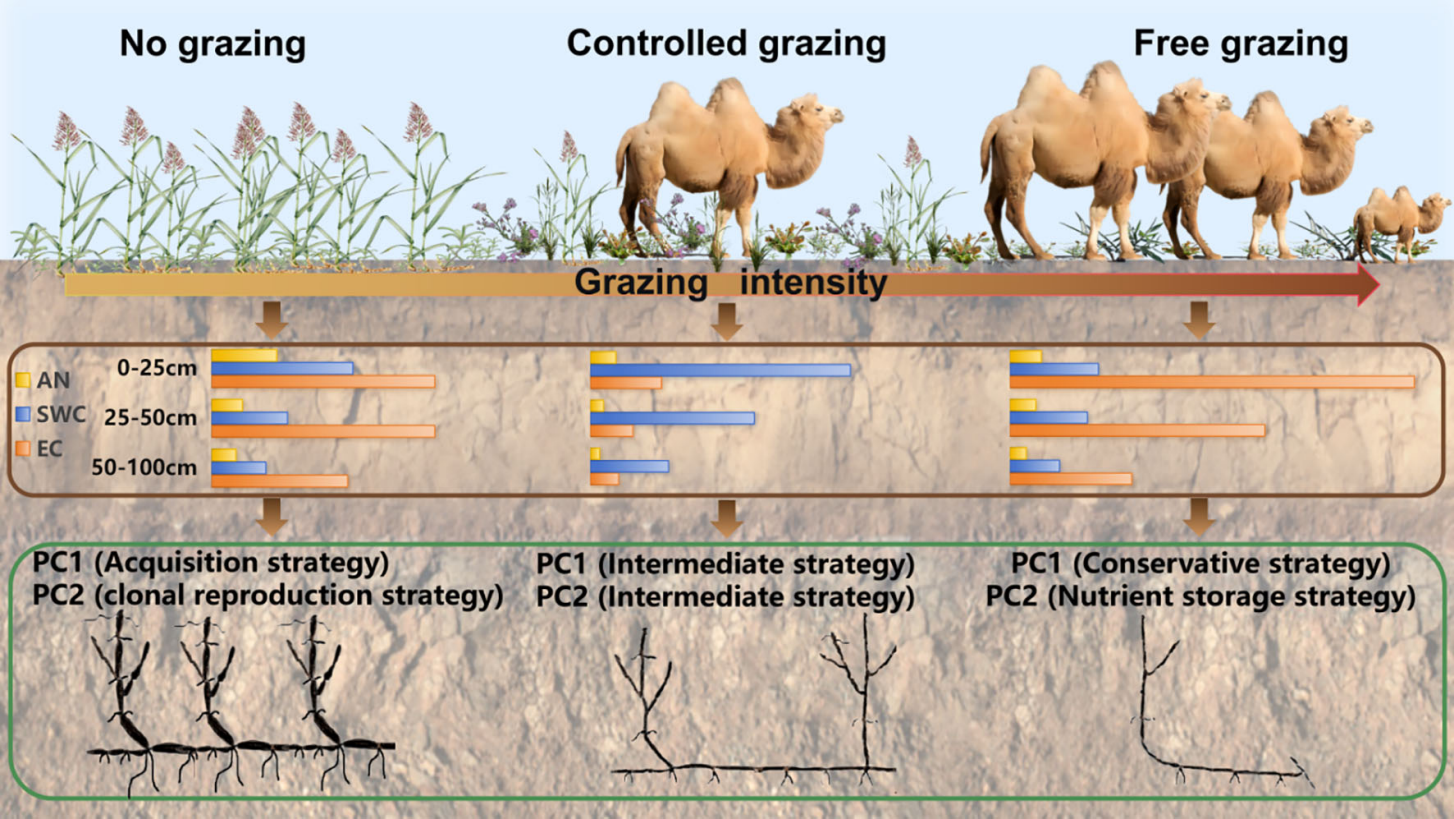
Mechanistic framework illustrating how camel grazing drives soil heterogeneity and shifts root economic strategies.

This study extends the theory of root economic strategies from natural environmental gradients to gradients of camel grazing disturbance (Díaz et al., 2016), confirming that the trade-off between “resource competition” and “disturbance tolerance” in plants follows similar economic logic, while emphasizing that this trade-off exhibits strong depth-dependence and nonlinear threshold characteristics (Le Bagousse-Pinguet et al., 2025). PCA-envfit analysis indicates that soil AN, EC, and SWC collectively explain 62.0%-73.4% of the variation in the spatial distribution of root traits (**Supplementary Table 3**), with the explanatory power of these three factors increasing in that order, providing site-specific ecological indicators for quantifying the strength of soil-subterranean organ coupling in this study system (Lachaise et al., 2022). From a management perspective, the quantitative thresholds proposed in this study provide site-specific ecological boundaries for precision grassland management in similar arid saline meadows: maintaining soil AN above 42 mg·kg^-1^ and controlling EC below 3200 µS cm^-1^ may be key objectives for sustaining the resource-acquisition functional trait syndrome of dominant species’ underground functional traits and soil carbon sequestration functions in this specific study system (**Figures 5**, **7**).

The “linear decrease” and “difference decrease” trends observed in the study are supported by statistical analyses. For example, the R² value for the linear decrease in SOC with grazing intensity is 0.85, indicating a strong linear relationship (He et al., 2025; Wu et al., 2024). The interaction effect between grazing intensity and soil depth on nutrient contents is also statistically significant (*P* < 0.05), consistent with findings that topsoil and subsoil respond differently to grazing disturbance (Li et al., 2025a; Ramesh et al., 2024). Regarding the “marked directional differences”, soil water content (SWC) shifts from decreasing to increasing when grazing intensity exceeds 1.5 camels/ha, a threshold that aligns with nonlinear responses reported in dryland grazing systems (Le Bagousse-Pinguet et al., 2025; Niu et al., 2025). The “filtering effect” becomes statistically insignificant below 50 cm depth, echoing observations that grazing impacts on soil properties attenuate with depth (Wu et al., 2022). The soil health thresholds defined in this context are EC > 3000 µS·cm^-^¹ and SOC < 10 g·kg^-^¹, beyond which the system shifts from “beneficial” to “salinity risk” status; such threshold dynamics are characteristic of state-and-transition models in rangelands (Briske et al., 2005; Rengasamy, 2010; Niu et al., 2025).

Camel trampling interacts with the unique physics of saline−alkali soils to promote carbonate accumulation. The high sodium content in saline−alkali soils disperses soil particles, increasing soil porosity (Rengasamy, 2010). Camel trampling compacts the soil, reducing porosity and promoting the precipitation of carbonate minerals (Xu et al., 2012). Camels possess broad, padded hooves and unique urination/defecation patterns that differ from other livestock (Faye, 2015; Kakar, 2025); these characteristics may exacerbate the observed effects. However, the “water retention” observed during compaction may not be sustainable. Prolonged compaction can lead to anaerobic conditions in the soil, inhibiting root respiration and nutrient uptake, and can also promote surface crusting, further impeding plant recovery (Briske et al., 2005; Niu et al., 2025). To address these issues, specific stocking rate guidelines are recommended. For arid saline meadows, a stocking rate of 0.5 camels/ha is optimal for maintaining soil health and plant productivity, a level that balances forage utilization with preservation of soil structure and function (Askar et al., 2024; Mohammed et al., 2020; Yan et al., 2024).

We acknowledge two methodological limitations. First, grazing intensity is ordinal rather than continuous; we addressed this using dummy coding in the OLS path analysis, but future studies with more treatment levels could enable continuous or nonlinear approaches. Second, unmeasured factors such as root exudates and microbial communities may also mediate grazing effects on soil properties and warrant further investigation (Shabtai et al., 2024; Song et al., 2022).

## 5 Conclusion

Camel grazing depletes nitrogen, accumulates salt, and redistributes water in grassland soils. These effects drive two functionally decoupled root trait shifts. Along the water-nitrogen axis, root economic strategies transition from an acquisitive to a conservative state, whereas along the salinity-nitrogen stress axis, rhizome economic strategies shift from clonal reproductive investment toward storage capacity. Grazing has no direct effect on PC2. The critical thresholds include SWC below 10%, EC above 3,144 µS·cm^-^¹, and AN below 42 mg·kg^-^¹. These findings support the moderate disturbance hypothesis and provide a scientific basis for precision grazing management based on soil trait thresholds. To maintain plant acquisition economic strategies, it is recommended to keep AN > 42 mg·kg^-^¹, EC < 3200μS·cm^-^¹; SWC > 10%. Moderate grazing intensity (CG) plays a unique role in maintaining belowground trait diversity and soil functional stability. It balances the need for forage utilization with the preservation of soil structure and function. It should be noted that these results are site-specific patterns associated with the grazed and ungrazed paddocks in this study, particularly along the distance-to-water gradient, rather than broadly generalizable causal effects of camel grazing.

## Data Availability

The original contributions presented in the study are included in the article/**Supplementary Material**. Further inquiries can be directed to the corresponding authors.
