# Supplementary material for: Camel grazing reshapes root and rhizome economic strategies and trade-offs: synergistic roles of soil water, salinity, and nitrogen availability
Source: Front Plant Sci. 2026 Jul 9;17:1876859. doi: 10.3389/fpls.2026.1876859 (PMC13391936; doi:10.3389/fpls.2026.1876859)
Supplement: Supplementary file 1 [file DataSheet1.docx]

Camel grazing reshapes root and rhizome economic strategies and trade-offs: synergistic roles of soil water, salinity, and nitrogen availability

Jing Zhang^1,2^, Kai Yan^2^, Wei Zheng^1,3*^, Yingzhi Gao^1,3*^, Meng Cui^4^, Jiashaer^2^, Yang Li^2^, Xiang Li^2^, Yerlan Akebieke^2^, Ayiding Hezhati^2^, Lianghong Wu^2^

^1^College of Grassland Science, Xinjiang Agricultural University, Urumqi, China

^2^Grassland General Station of Xinjiang Uygur Autonomous Region, Urumqi, China

^3^Xinjiang Key Laboratory of Grassland Resources and Ecology, Urumqi, China

^4^College of Grassland Science, Inner Mongolia Agricultural University, Hohhot, China

^5^Xinjiang Uygur Autonomous Region Grassland Biological Disaster Prevention and Control Center (Regional Wildlife Protection and Monitoring Center), Urumqi, China

*** Correspondence:**Corresponding Authors
[zw065@126.com,](mailto:zw065@126.com,) [gaoyz@nenu.edu.cn](mailto:gaoyz@nenu.edu.cn)

# Supplementary Figure


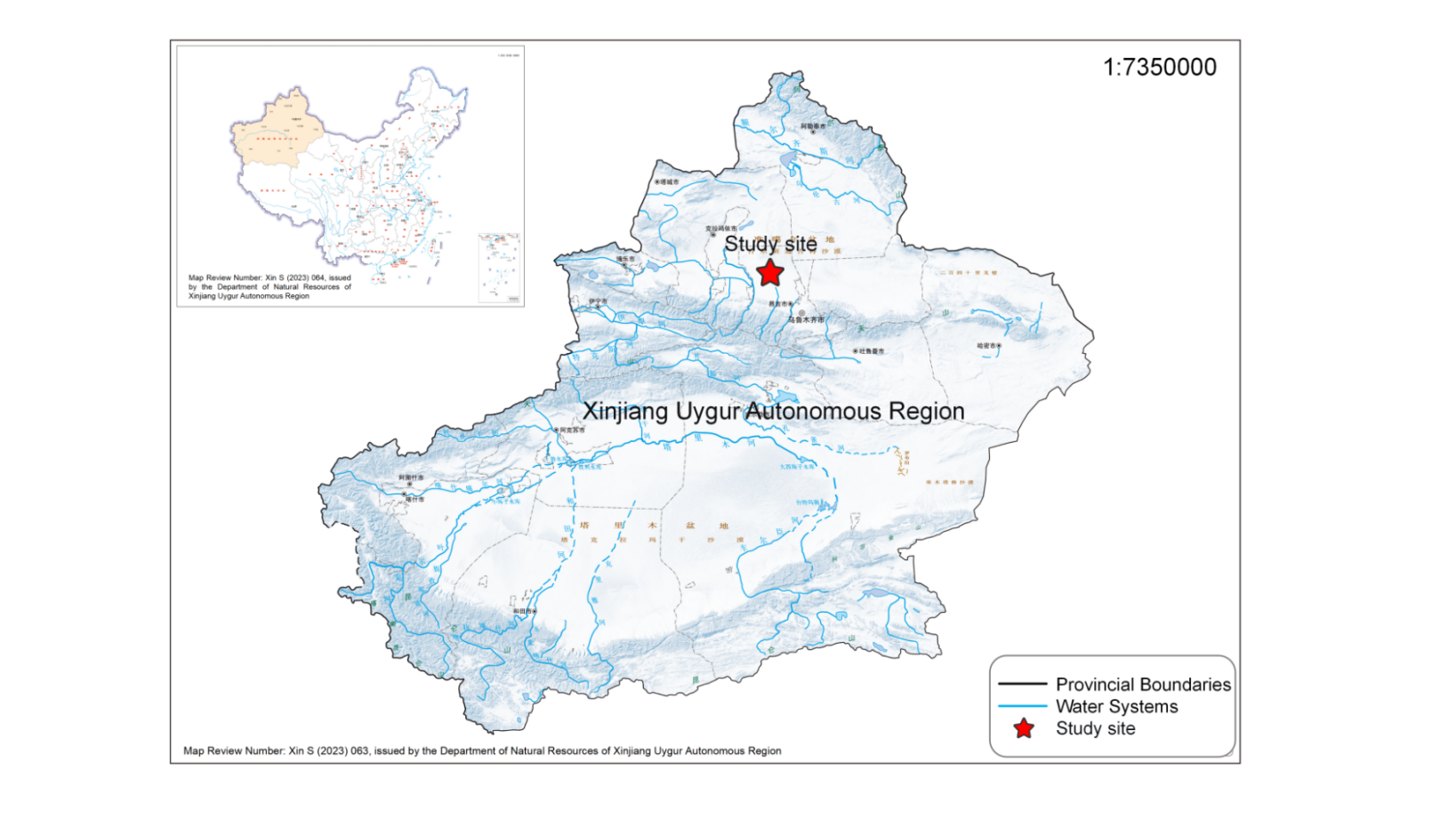


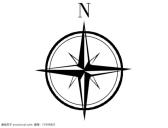


**Supplementary Figure 1.** Geographic Location of the Study Area. Located in Xiasangong Village, Yuanhucun Town, Hutubi County, Xinjiang Uygur Autonomous Region, China.

# Supplementary Tables

**Supplementary Table 1.** Meteorological data of the study area from 2020 to 2024. The data is sourced from the Hutubi County Meteorological Bureau of the Xinjiang Uygur Autonomous Region.

| Year | Mean annual precipitation  (mm) | Mean annual temperature   (℃) | Mean annual  minimum temperature   (℃) | Mean annual  maximum temperature   (℃) |
| --- | --- | --- | --- | --- |
| 2020 | 78.50 | 9.00 | -24.30 | 37.40 |
| 2021 | 189.50 | 8.50 | -29.10 | 40.30 |
| 2022 | 135.50 | 9.20 | -26.30 | 40.00 |
| 2023 | 157.00 | 9.20 | -29.70 | 40.90 |
| 2024 | 210.70 | 8.80 | -28.10 | 39.70 |

# Supplementary Table 2. Results of permutational multivariate analysis of variance (PERMANOVA) based on permutation tests. *Df*, degrees of freedom; SumOfSqs, sum of squares; *R*^2^, coefficient of determination (proportion of variance explained by each component); *F*, *F*-statistic; *P*-value, significance probability obtained from permutation tests with 999 (or specified number of) permutations. *** *P*<0.001.

|  | Df | SumOfSqs | *R*^2^ | *F*-value | *P*-value | Significance |
| --- | --- | --- | --- | --- | --- | --- |
| Model | 2 | 8.24 | 0.85 | 168.76 | 0.001 | *** |
| Residual | 62 | 1.51 | 0.16 |  |  |  |
| Total | 64 | 9.75 | 1 |  |  |  |

**Supplementary Table 3.** Loadings of root and rhizome functionnal traits on the first two principal components (PC1 and PC2). PC1 loading and PC2 loading represent the correlation coefficients between each trait and the principal components, with larger absolute values indicating greater contributions. PC1 contrib and PC2 contrib are the squared loadings, representing the contribution of each trait to the variance explained by PC1 and PC2; Total contrib is the sum of these two contributions, reflecting the overall importance of each trait to the first two principal components. PC1 and PC2 explained 58.8% and 12.9% of the total variance, respectively. Positive loadings on PC1 are mainly associated with conservative strategy-related traits, including root biomass (RZB), root length (RZL), and root surface area (RZSA). Negative loadings on PC1 are mainly associated with acquisitive strategy-related traits. Positive loadings on PC2 are mainly associated with reproduction-related traits.

| Trait | PC1 loading | PC2 loading | PC1 contrib | PC2 contrib | Total contrib |
| --- | --- | --- | --- | --- | --- |
| RZB2 | 0.24 | -0.13 | 0.06 | 0.02 | 0.07 |
| RZL2 | 0.23 | 0.00 | 0.06 | 0.00 | 0.06 |
| RZB3 | 0.23 | -0.12 | 0.05 | 0.01 | 0.07 |
| RZSA1 | 0.23 | -0.07 | 0.05 | 0.00 | 0.06 |
| RZB1 | 0.23 | 0.11 | 0.05 | 0.01 | 0.06 |
| RZSA2 | 0.23 | -0.15 | 0.05 | 0.02 | 0.08 |
| RL3 | 0.23 | -0.03 | 0.05 | 0.00 | 0.05 |
| RV3 | 0.22 | -0.05 | 0.05 | 0.00 | 0.05 |
| RZSA3 | 0.22 | -0.19 | 0.05 | 0.03 | 0.08 |
| RV2 | 0.22 | 0.13 | 0.05 | 0.02 | 0.07 |
| RZV2 | 0.22 | -0.21 | 0.05 | 0.04 | 0.09 |
| RZV3 | 0.22 | -0.24 | 0.05 | 0.06 | 0.10 |
| RZL3 | 0.20 | 0.02 | 0.04 | 0.00 | 0.04 |
| RV1 | 0.20 | 0.18 | 0.04 | 0.03 | 0.07 |
| RZL1 | 0.20 | 0.18 | 0.04 | 0.03 | 0.07 |
| RZD2 | 0.20 | -0.23 | 0.04 | 0.05 | 0.09 |
| RSA2 | 0.19 | 0.30 | 0.04 | 0.09 | 0.13 |
| RSA3 | 0.19 | 0.22 | 0.04 | 0.05 | 0.09 |
| RSA1 | 0.18 | 0.28 | 0.03 | 0.08 | 0.11 |
| RZD3 | 0.17 | -0.30 | 0.03 | 0.09 | 0.12 |
| RL2 | 0.15 | 0.28 | 0.02 | 0.08 | 0.10 |
| SRzL1 | -0.14 | -0.14 | 0.02 | 0.02 | 0.04 |
| SRzL2 | -0.13 | -0.08 | 0.02 | 0.01 | 0.02 |
| SRzL3 | -0.11 | -0.04 | 0.01 | 0.00 | 0.01 |
| RL1 | 0.10 | 0.35 | 0.01 | 0.12 | 0.13 |
| RZD1 | 0.10 | -0.34 | 0.01 | 0.12 | 0.13 |

**Supplementary Table 4.** Vector fitting results (envfit) of environmental factors with ordination axes (PC1, PC2). PC1 and PC2 are the first two ordination axes; the direction cosines (PC1, PC2 columns) indicate the direction of the environmental factor vectors in the ordination space; R^2^ is the coefficient of determination, representing the proportion of variance of the environmental factor explained by the ordination axes (goodness-of-fit); Pr(>r) is the significance probability obtained from permutation tests with 999 permutations. Significance codes: *** *P*<0.001, ** *P*<0.01, * P<0.05. Permutation type: free permutation.

| Variable | PC1 | PC2 | *R*^2^ | *P*-value | Significance |
| --- | --- | --- | --- | --- | --- |
| SWC | -0.31 | -0.95 | 0.62 | 0.001 | *** |
| EC | -0.21 | -0.98 | 0.69 | 0.001 | *** |
| AN | 0.57 | -0.82 | 0.73 | 0.001 | *** |

**Supplementary Table 5.** Nonlinear response thresholds of root economic strategy to soil water content (SWC), electrical conductivity (EC), and available nitrogen (AN). ΔAIC represents the improvement in AIC value of the piecewise model compared to the linear model.

| Variable | Unit | Threshold | GAM *R*²(adj) | Deviance explained (%) | ΔAIC | *P*-value |
| --- | --- | --- | --- | --- | --- | --- |
| SWC | % | 12.14 | 0.93 | 93.50% | -69.00 | 1.35e-15 |
| EC | µS·cm^-1^ | 3197.13 | 0.75 | 77.80% | -38.10 | 2.70e-09 |
| AN | mg·kg^-1^ | 42.03 | 0.84 | 85.60% | -22.40 | 4.19e-06 |

**Supplementary Table 6.** Standardized path coefficients, bootstrap standard errors, p-values, coefficients of determination (*R*²), and variance inflation factors (VIF) for the path analysis. Grazing intensity was treated as a categorical variable with no grazing (NG) as the reference, generating two dummy variables: CG (0.5 camels/ha) and FG (2 camels/ha). Standardized path coefficients (*β*) are presented along with standard errors obtained from 1,000 bootstrap resamples, as well as 2.5% and 97.5% percentile confidence intervals.***P* < 0.01, ****P* < 0.001; ns, not significant. *R*² values are the coefficients of determination for the respective response variables (SWC, EC, AN, PC1, PC2). VIF is provided only for the equations predicting PC1 and PC2, where multiple predictors exist; all VIF values are below 2, indicating no multicollinearity. For the SWC, EC, and AN equations, each has only two dummy variables as predictors, so VIF is not applicable (NA).

| Predictor Variable | Response Variable | Estimate | Std.Error | *R*^2^ | VIF | *P*-value | Significance |
| --- | --- | --- | --- | --- | --- | --- | --- |
| CG | SWC | -0.32 | 0.04 | 0.35 | / | <0.001 | *** |
| FG | SWC | 0.39 | 0.04 | 0.35 | / | <0.001 | *** |
| CG | EC | -0.33 | 0.03 | 0.58 | / | <0.001 | *** |
| FG | EC | 0.57 | 0.03 | 0.58 | / | <0.001 | *** |
| CG | AN | -0.56 | 0.04 | 0.59 | / | <0.001 | *** |
| FG | AN | -0.79 | 0.03 | 0.59 | / | <0.001 | *** |
| SWC | PC1 | 0.01 | 0.03 | 0.61 | 1.42 | 0.76 | ns |
| EC | PC1 | 0.34 | 0.03 | 0.61 | 1.47 | <0.001 | *** |
| AN | PC1 | -0.63 | 0.02 | 0.61 | 1.05 | <0.001 | *** |
| SWC | PC2 | -0.13 | 0.04 | 0.40 | 1.42 | 0.003 | ** |
| EC | PC2 | -0.49 | 0.04 | 0.40 | 1.47 | <0.001 | *** |
| AN | PC2 | -0.43 | 0.03 | 0.340 | 1.05 | <0.001 | *** |
